# Cleanliness

**Published:** 1897-08

**Authors:** 


### Cleanliness.

Cleanliness of what? Why, of everything. Of the physical, the intellectual and the spiritual; but it is not all of these, that will now claim attention, but only a small fraction of one viz, the first.

The personal cleanliness of the dentist has been discussed somewhat, but it is not this that we will talk about now. The dental office and its appertinances, has been pretty fully discussed, and with fairly good results; neither is it the instruments used by the dentist that will now receive attention. It is nothing less, nor more than the mouth of the patient.

What, the mouth of our patients unclean? We will ask every dentist to answer this question for himself. My good brother in full practice; how many of those who apply to you for professional service do you find with teeth and mouth clean, free from impurities, and the soft tissues in a healthy condition? How many are found with the teeth free from deposits of some kind or other? Deposits or stains that render the teeth unsightly, and tend to produce disease, in either the teeth or soft tissues, or both. It is more particularly to the impurities on the teeth that I would now call attention, not that they are not seen, but that they are seen and neglected, how often is it that teeth are filled, and the patient dismissed with stain, and too often with masses of salivary calculus upon them which renders the teeth unsightly and the mouth impure, if not diseased. Fillings in unclean teeth are much less likely to be permanent than in those free from impurities; and furthermore fillings are more likely to be well made in clean then in dirty teeth.

Then let it be the practice, never to be omitted, to clean every tooth, that is to receive a filling as perfectly as possible before the latter operation, and then impress upon the patient the importance of keeping the tooth or teeth in that condition. Every patient that is disposed to neglect the teeth in respect to cleanliness, should visit the dentist every few weeks, for a lecture if nothing more.

The dentist cannot afford to assume the responsibility of a patient's teeth, who will not conform to the instructions of the dentist in regard to the care of his teeth.

